# Secreted in a Type III Secretion System-Dependent Manner, EsaH and EscE Are the Cochaperones of the T3SS Needle Protein EsaG of Edwardsiella piscicida

**DOI:** 10.1128/mbio.01250-22

**Published:** 2022-07-21

**Authors:** Zhi Xiong Zeng, Lu Yi Liu, Shui Bing Xiao, Jin Fang Lu, Ying Li Liu, Jing Li, Yuan Ze Zhou, Li Jing Liao, Duan You Li, Ying Zhou, Pin Nie, Hai Xia Xie

**Affiliations:** a College of Bioengineering, Qilu University of Technology, Jinan, Shandong Province, China; b State Key Laboratory of Freshwater Ecology and Biotechnology, Institute of Hydrobiology, Chinese Academy of Sciences, Wuhan, Hubei Province, China; c College of Life Science and Technology, Huazhong Agricultural University, Wuhan, Hubei Province, China; d School of Laboratory Medicine and Life Sciences, Wenzhou Medical University, Wenzhou, Zhejiang Province, China; e School of Marine Science and Engineering, Qingdao Agricultural University, Qingdao, Shandong Province, China; f Laboratory for Marine Biology and Biotechnology, Pilot National Laboratory for Marine Science and Technology, Qingdao, Shandong Province, China; University of Michigan—Ann Arbor

**Keywords:** cochaperone, secretion, T3SS needle protein, *Edwardsiella piscicida*

## Abstract

The intracellular EscE protein tightly controls the secretion of the type III secretion system (T3SS) middle and late substrates in Edwardsiella piscicida. However, the regulation of secretion by EscE is incompletely understood. In this work, we reveal that EscE interacts with EsaH and EsaG. The crystal structures of the EscE-EsaH complex and EscE-EsaG-EsaH complex were resolved at resolutions of 1.4 Å and 1.8 Å, respectively. EscE and EsaH form a hydrophobic groove to engulf the C-terminal region of EsaG (56 to 73 amino acids [aa]), serving as the cochaperones of T3SS needle protein EsaG in *E. piscicida*. V61, K62, M64, and M65 of EsaG play a pivotal role in maintaining the conformation of the ternary complex of EscE-EsaG-EsaH, thereby maintaining the stability of EsaG. An *in vivo* experiment revealed that EscE and EsaH stabilize each other, and both of them stabilize EsaG. Meanwhile, either EscE or EsaH can be secreted through the T3SS. The secondary structure of EsaH lacks the fourth and fifth α helices presented in its homologs PscG, YscG, and AscG. Insertion of the α4 and α5 helices of PscG or swapping the N-terminal 25 aa of PscG with those of EsaH starkly decreases the protein level of the chimeric EsaH, resulting in instability of EsaG and deactivation of the T3SS. To the best of our knowledge, these data represent the first reported structure of the T3SS needle complex of pathogens from Enterobacteriaceae and the first evidence for the secretion of T3SS needle chaperones.

## INTRODUCTION

The type III secretion system (T3SS) is a protein secretion machinery that delivers virulence effectors from the cytosol of a variety of Gram-negative pathogens directly into the host cell cytoplasm. The injectisome is composed of membrane-embedded protein rings extended by a hollow needle formed by a single polymerized shaft, through which effectors are secreted or translocated to aid infection.

Edwardsiella piscicida, previously known as Edwardsiella tarda (1), is a Gram-negative intracellular pathogen and one of the causes of hemorrhagic septicemia in fish. It is also an emerging agent for human infection through wound contact with ill fish (2). The T3SS of *E. piscicida* is encoded by its chromosome and is required for its survival and replication in epithelial cells or macrophage cells (3–5). Of the substrates secreted by the T3SS, EseB, EseC, and EseD function as the translocon, forming pores on host membranes through which effectors are translocated (6). EseG, EseJ, EseH, and EseK have been identified to be the T3SS effectors of *E. piscicida* (7–10). Besides the translocon and effectors, needle protein is also secreted through the T3SS basal body. The T3SS needle of *Yersinia*, Pseudomonas, *Aeromonas*, enteropathogenic Escherichia coli (EPEC), *Shigella*, Salmonella pathogenicity island 2 (SPI2), Salmonella SPI1, *Burkholderia*, or Chlamydia trachomatis is composed of a single protein: YscF, PscF, AscF, EscF, MxiH, SsaG, PrgI, BsaL, or CdsF, respectively (11–21). Chaperones are required to bind to newly synthesized needle protein and to keep it in a secretion-competent state (22, 23). The sequence homologies between type III needle chaperones are notably low among different bacterial pathogens; however, they share a similar structure and possess common features, such as their small size and an often acidic pI. Before secretion, chaperones bind to the C-terminal region of needle protein to maintain its soluble state and to prevent it from undergoing self-assembly or degradation by YscE-YscG in *Yersinia* spp., PscE-PscG in Pseudomonas aeruginosa, AscE-AscG in Aeromonas hydrophila, EscE-EscG in EPEC, and CdsE-CdsG in C. trachomatis (12, 16, 18, 21, 24–26). Among them, PscE-PscG was first discovered and characterized in P. aeruginosa: PscE and PscG were shown to form a stable, soluble complex with PscF in the cytoplasm at a 1:1:1 ratio, thus blocking premature polymerization of PscF (24, 27).

Chaperones for the type III needle of *E. piscicida* remain unidentified. Our recent study demonstrated that the type III protein EscE (Orf13 protein) shares sequence homology with SsaE in Salmonella enterica, YscE in Yersinia enterocolitica, and CV2595 in Chromobacterium violaceum (28). YscE and YscG are the cochaperones of T3SS needle YscF. However, we failed to find a homolog of YscG in the *E. piscicida* T3SS gene cluster through a primary sequence homology search. In this study, the proteins in the EscE-EsaH complex are proven to be the cochaperones of T3SS needle protein EsaG, as revealed by its crystal structure. Moreover, it is demonstrated that both EscE and EsaH are secreted in a T3SS-dependent manner.

## RESULTS

### Sequence analysis of EsaG, EsaH, and EscE.

**FIG 1:**
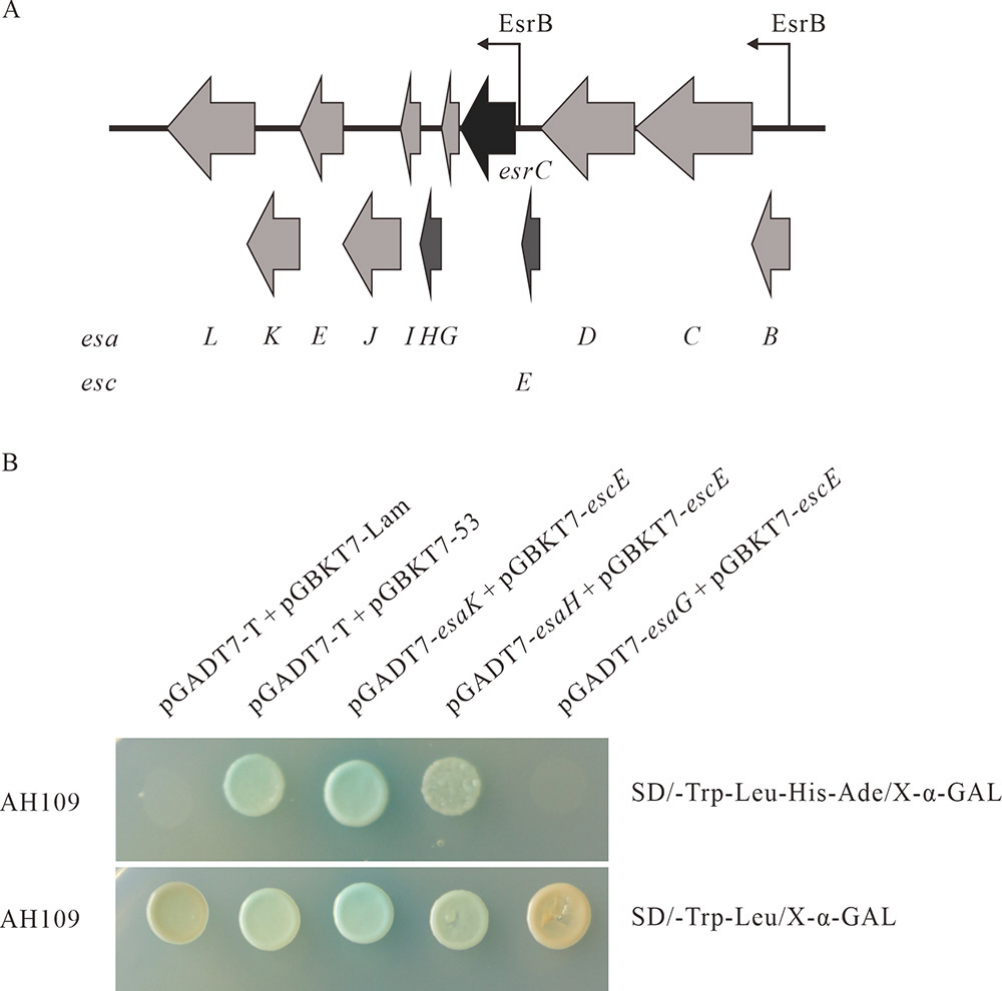
Interaction of EscE with EsaH. (A) Schematic representation of the *escE-esaL* region of the *E. piscicida* T3SS. Arrows represent each of the open reading frames. (B) Interaction of EscE with EsaH and EsaK as revealed by yeast two-hybrid assay results. Yeast two-hybrid results were obtained using high-stringency Sabouraud dextrose lacking Trp, Leu, His, and Ade (SD/−Trp −Leu −His −Ade) (top) and medium-stringency SD/−Trp −Leu (bottom). Yeast strains transformed with pGADT7-*esaH* plus pGBKT7-*escE* or pGADT7-*esaK* plus pGBKT7-*escE* were able to grow on the selective high-stringency plate as blue colonies like the positive control (transformed with pGADT7-T plus pGBKT7-53). Yeast strains transformed with pGADT7-T plus pGBKT7-Lam were the negative control.

The *esaG* gene is located upstream of *esaH* and downstream of *esrC* in the T3SS gene cluster of *E. piscicida* (Fig. 1A). It encodes a peptide of 73 amino acids (aa) with a predicted molecular mass of 8.0 kDa and a pI of 6.5. Using CDD (http://www.ncbi.nlm.nih.gov/Structure/cdd/wrpsb.cgi) and InterPro (http://www.ebi.ac.uk/interpro/search/sequence) for informatic analysis, EsaG was found to belong to the T3SS_needle_F superfamily and shares homology with the type III needle protein MxiH of *Shigella*, YscF of Yersinia pestis, SsaG of Salmonella enterica, PscF of P. aeruginosa, and EscF of EPEC. Notably, EsaG shares 61.8% and 64.4% similarity with SsaG and EscF, respectively, and 42.6%, 43.0%, and 45.3% similarity with YscF, AscF, and PscF, respectively (Table 1).

**TABLE 1:** Similarity matrix for EscE, EsaG, and EsaH homologs

*E. piscicida* protein	Homologous protein (% similarity)^*a*^				
	*S. enterica* (SPI-2)	EPEC/eHEC	*Y. enterocolitica*	*A. hydrophila*	*P. aeruginosa*
EscE	SsaE (36.6)	EscE_ec_ (41.9)	YscE (39.2)	AscE (42.9)	PscE (35.1)
EsaG	SsaG (61.8)	EscF (64.4)	YscF (42.6)	AscF (43.0)	PscF (45.3)
EsaH	SsaH (46.4)	EscG (35.5)	YscG (25.0)	AscG (16.8)	PscG (7.7)

a Protein sequences of EscE, EsaG, and EsaH and their homologs in other bacteria were compared. The percentage of similar amino acid residues was calculated using EMBOSS Needle protein alignment (www.ebi.ac.uk/Tools/psa/emboss_needle/). The corresponding protein sequences were retrieved through the GenBank accession numbers for the proteins EscE (WP_012847727.1), SsaE (NP_460361.1), EscE_ec_ (WP_000628726.1),YscE (CAF25424.1), AscE (ANT67527.1), PscE (NP_250409.1), EsaG (ACY83705.1), SsaG (NP_460371.1), EscF (NP_312579.1), YscF (CAF25425.1), AscF (WP_021229954.1), PscF (NP_250410.1), EsaH (ADM40922.1), SsaH (BCH84983.1), EscG (NP_312578.1), YscG (WP_011901822.1), AscG (ABF70178.1), and PscG (NP_250411.1).

The *esaH* gene is located between *esaG* and *esaI* in the *esrC*-*esaL* transcriptional unit in the T3SS gene cluster of *E. piscicida* (Fig. 1A). The *esaH* gene encodes a peptide of 88 amino acids, with a predicted molecular mass of 9.6 kDa and a pI of 4.9. Using CDD and InterPro, EsaH was found to be in the same family as the Salmonella SPI2 protein SsaH, which recently has been reported to be one of the chaperones of the Salmonella SPI2 needle protein SsaG, as revealed by a coimmunoprecipitation assay (17). EsaH shares 46.4% similarity with S. enterica SsaH, 35.5% similarity with EPEC/enterohemorrhagic *E. coli* (EHEC) EscG, 25% similarity with Y. enterocolitica YscG, 16.8% similarity with A. hydrophila AscG, and 7.7% similarity with P. aeruginosa PscG (Table 1).

The *escE* gene (previously known as *orf13*) is located upstream of *esaG*, spaced by *esrC* in the T3SS gene cluster of *E. piscicida* (Fig. 1A). It encodes an 8.7-kDa protein with a pI of 7.9. EscE belongs to the T3SS YscE superfamily. When domains were searched using Delta-BLAST, EscE was found to share 36.6%, 35.1%, 39.2%, and 42.9% similarity with SsaE of Salmonella spp., PscE of P. aeruginosa, YscE of Y. pestis, and AscE of A. hydrophila, respectively (Table 1). Unexpectedly, our previous study revealed that EscE can be secreted and translocated in a T3SS-dependent manner, and it regulates the secretion of the T3SS proteins according to its own steady-state protein level inside *E. piscicida* (28). Accordingly, it was verified that EscE (Orf13) is secreted and directly interacts with mammalian factor Cugbp2 upon its translocation (29).

### EscE interacts with EsaH and EsaK.

To search for the interacting partner of EscE, we cloned *escE* into the yeast two-hybrid system vector pGBKT7 and explored the pairwise interaction between EscE and 23 proteins of the *E. piscicida* T3SS, as described by Liu et al. (30). The results showed that yeast strains transformed only with pGBKT7-*escE* plus pGADT7-*esaK* or pGADT7-*esaH* were able to grow in the selective medium as blue colonies, similar to the positive control, in which the yeast was transformed with pGBKT7-53 plus pGADT7-T. The yeast strain transformed with pGBKT7-Lam and pGADT7-T was used as the negative control, which failed to grow in the selective medium (Fig. 1B). Yeast strains transformed with pGBKT7-*escE* and the other 21 recombinant plasmids did not grow (see Fig. S1 in the supplemental material). This result indicates that either EsaH or EsaK may interact with EscE.

10.1128/mbio.01250-22.1 FIG S1 Yeast strains transformed with pGBKT7-*escE* plus pGADT7 with insertions of *esaC*, *esaD*, *esaJ*, *esaB*, *esaI*, *esaL*, *esaN*, *esaR*, *esaS*, *esaT*, *esaU*, *orf1B*, *esaE*, *orf26*, *escE*, *eseB*, *eseC*, *eseD*, *eseE*, *esrB*, or *esrC* failed to grow on the SD/–Trp –Leu –His –Ade plate. Download FIG S1, DOC file, 2.1 MB. Copyright © 2022 Zeng et al. 2022 Zeng et al. https://creativecommons.org/licenses/by/4.0/ This content is distributed under the terms of the Creative Commons Attribution 4.0 International license.

### Crystal structure of the EscE-EsaH complex.

**FIG 2:**
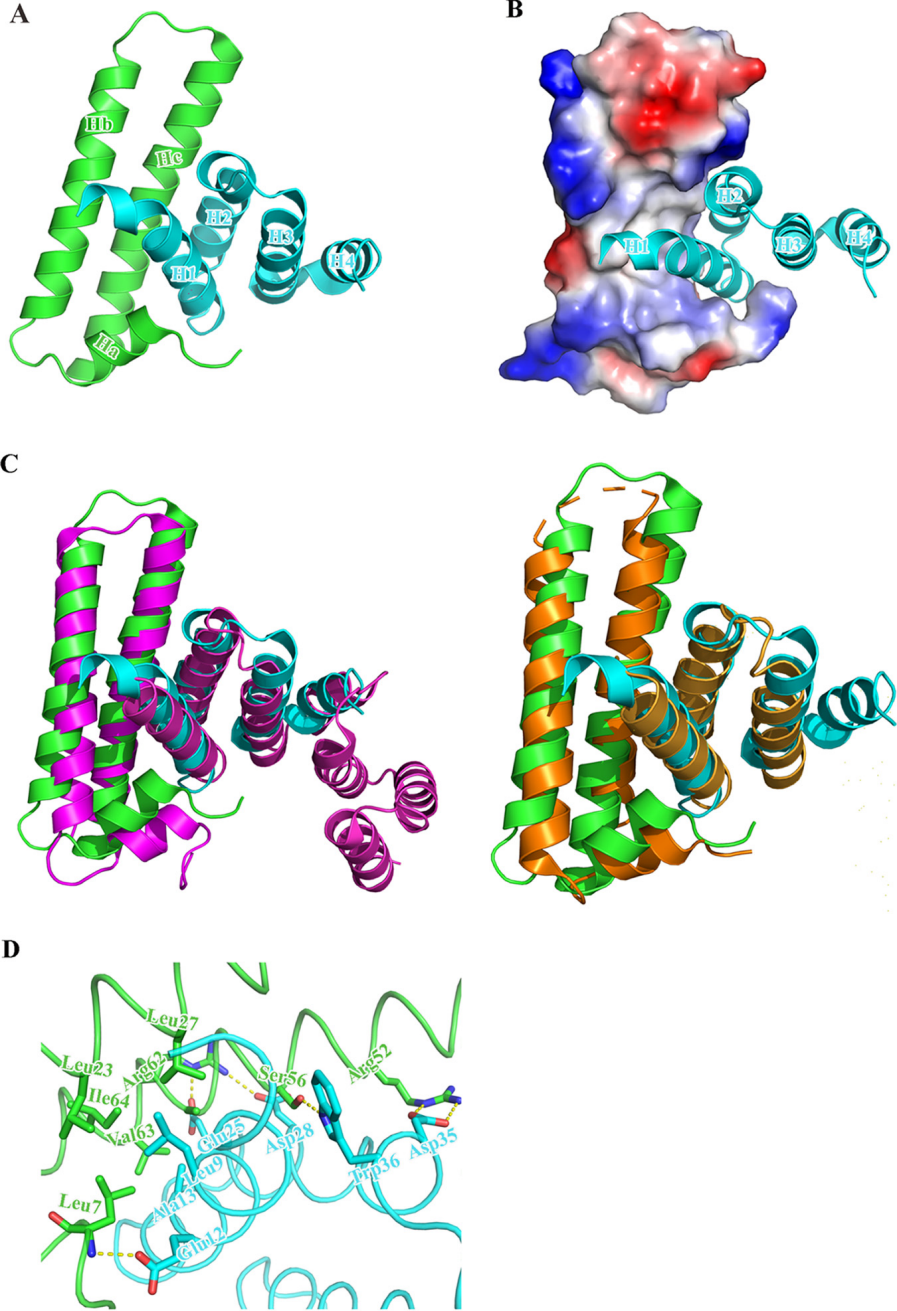
Structure of the EsaH-EscE complex. (A) Overall structure of the EsaH-EscE complex (cartoon), with the secondary structures labeled. EscE and EsaH are colored in green and cyan, respectively. (B) Electrostatic surface potential of the EsaH binding site of EscE, with EscE shown on the electrostatic surface and EsaH in the cartoon; (C) Superimposed Edwardsiella piscicida EsaH-EscE complex with Pseudomonas aeruginosa PscE-PscG complex (magenta) and Aeromonas hydrophila AscE-AscG complex (orange); (D) The view of EsaH-EscE interaction interface, in which the hydrogen bonding interactions are denoted by yellow dashed lines.

When expressed in E. coli BL21(DE3), EscE is soluble and stable, while EsaH is insoluble and unstable. By coexpression and copurification, we obtained the EscE-EsaH complex. The crystal structure of the EscE-EsaH complex was solved by single-wavelength anomalous diffraction (SAD) at a resolution of 1.8 Å, and it has been refined to an *R*_work_/*R*_free_ of 20%/22.8% with good geometry (Table 2). The final structure of EscE-EsaH consists of residues 5 to 73 of EscE and residues 2 to 68 of EsaH. The C-terminal region of EsaH (residues 69 to 88) is invisible, and no electron density could be traced in our structure, indicating that this region is flexible in this binary complex. However, EscE and EsaH form a compact fold, and the first helix of EsaH docked deeply into the concave surface formed by the two antiparallel helices (Hb and Hc) and the short helix (Ha) of EscE (Fig. 2A and B). Despite the low sequence homology, the triple-helix structure of EscE resembles that of AscE and PscE, with root mean square deviations (RMSDs) of 0.9 Å and 1.6 Å, respectively. As AscE-AscG and PscE-PscG are cochaperones, the EscE-EsaH complex was superimposed with AscE-AscG and PscE-PscG, and their structural similarity was revealed with RMSDs of 2.2 Å and 2.4 Å, respectively (Fig. 2C). Further inspection showed that Leu9 and Ala13 of EsaH and Leu7, Leu23, Leu27, V63, and Ile64 of EscE form a hydrophobic interface (Fig. 2D). Besides, the extensive hydrogen bonds (EsaH^Glu12^-EscE^Leu7^, EsaH^Glu25^-EscE^Arg62^, EsaH^Asp28^-EscE^Arg62^, EsaH^Trp36^-EscE^Ser56^, and EsaH^Asp35^-EscE^Arg52^) mediate the interactions between EsaH and EscE (Fig. 2D). The extensive interaction interface between EsaH and EscE makes EsaH stable in solution.

**TABLE 2:** Crystal data collection and refinement statistics

Parameter	Result for^*a*^:	
	EscE-EsaH (Se)	EscE-EsaH-EsaG
PDB ID	7Y6B	7Y6C
Data collection statistics		
Wavelength (Å)	0.9785	0.9791
Space group	P21	C2
Cell dimensions		
*a*, *b*, *c* (Å)	55.55, 52.75, 65.51	123.85, 57.75, 54.8
α, β, γ (°)	90.0, 114.0, 90.0	90.0, 98.8, 90.0
Resolution (Å)	1.8	1.4
*R*_merge_ (%)	6.7 (38.0)	5.3 (27.5)
*I*/σ〈*I*〉	9.0 (2.8)	14.8 (4.6)
Completeness (%)	97.6 (98.0)	98.1 (98.3)
Refinement statistics		
Resolution (Å)	39.6–1.8	37.8–1.4
No. of reflections	30,376	73,628
*R*_work_/*R*_free_ (%)	20.0/22.8	20.4/23.2
No. of atoms		
Protein	2,244	2,703
Water	114	277
B-factors (Å^2^)		
Protein	20.9	19.2
Water	19.3	28.4
RMSD		
Bond length (Å)	0.007	0.005
Bond angle (°)	0.804	0.779
Ramanchandran plot (%)		
Favored regions	99.28	100
Allowed regions	100	100
Outlier regions	0	0

a The highest-resolution shell is shown in parentheses.

### Both EscE and EsaH are required for the presence of EsaG.

**FIG 3:**
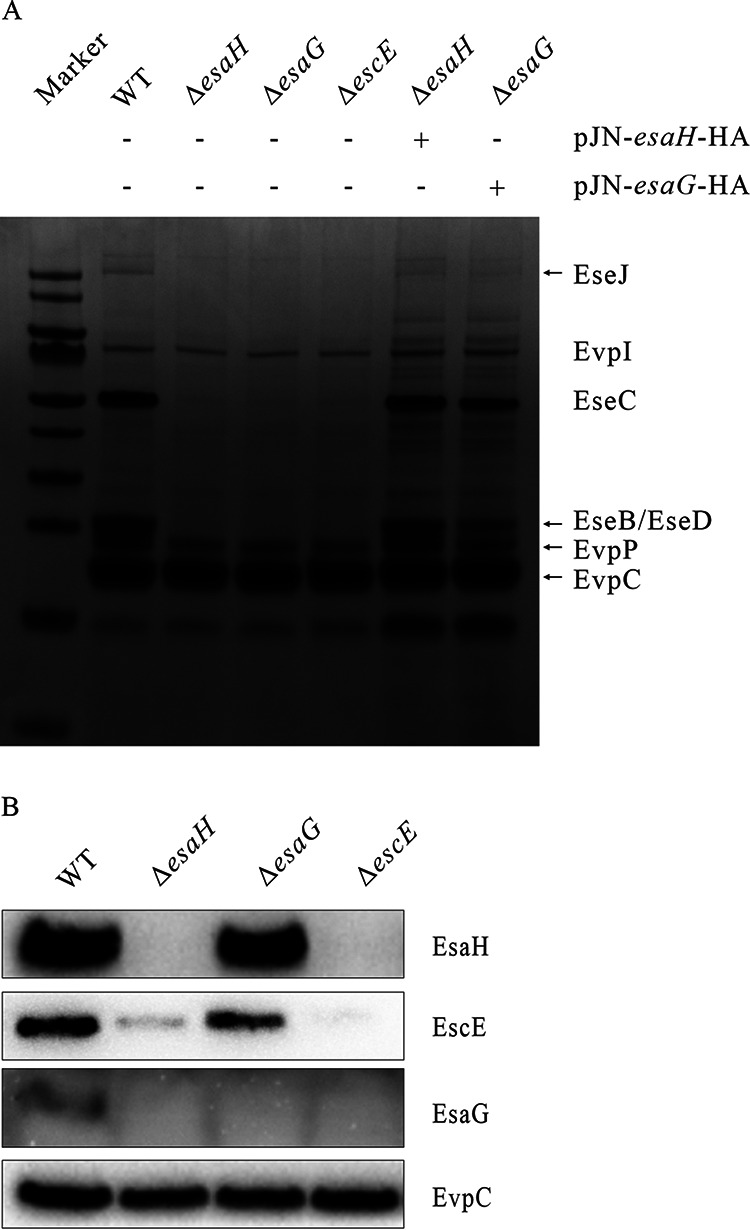

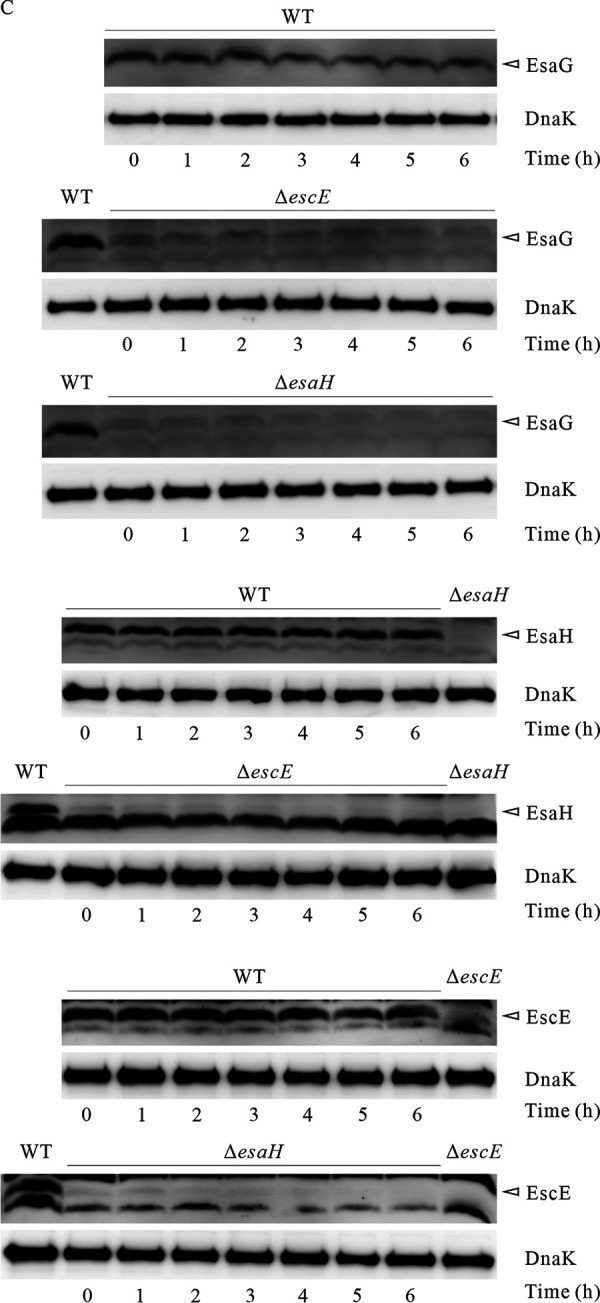
Importance of EscE, EsaH, and EsaG for the activity of the T3SS. (A) Protein secretion profiles of the *E. piscicida* wild-type (WT) and Δ*escE*, Δ*esaH*, Δ*esaG*, Δ*esaH*/pJN-*esaH*-HA, and Δ*esaG*/pJN-*esaG*-HA strains. Proteins in supernatants were concentrated and analyzed by Coomassie staining of 12% SDS-PAGE gel. (B) Steady-state protein levels of EscE, EsaH, and EsaG examined in the cell lysate of *E. piscicida* strains. Proteins on PVDF membranes were probed with anti-EscE, anti-EsaH, anti-EsaG, and anti-EvpC antibodies. EvpC, a protein secreted by the type VI secretion system (T6SS) but not by the T3SS, was used as a loading control. (C) EscE and EsaH stabilize each other, and both are required for maintaining the stability of EsaG. The *E. piscicida* WT 3×FLAG::*esaG* strain, Δ*escE* 3×FLAG::*esaG* strain, and Δ*esaH* 3×FLAG::*esaG* strain were cultured in the presence of 200 μg/mL Cm for different times, as indicated, and the bacterial pellets sampled were subjected to immunoblotting with FLAG (EsaG), EsaH, EscE, and DnaK antibodies. DnaK, a chaperone protein of *E. piscicida*, was used as a loading control. The immunoblotting data shown are representative images from three independent experiments.

EscE-EsaH resembles AscE-AscG and PscE-PscG, which are cochaperones of the T3SS needle protein. The T3SS needle protein in *E. piscicida* is EsaG (Table 1). Needle protein is the early substrate of the T3SS, and its failure in secretion always blocks secretion of the middle (translocon) and late (effector) substrates, as is the case for SPI2 of S. enterica (17) and LEE of EPEC (31). To investigate this in *E. piscicida*, the extracellular protein profiles of wild-type (WT) and Δ*esaG*, Δ*escE*, and Δ*esaH* mutant strains were compared. It was observed that disruption of EsaH, EscE, or EsaG obstructed the secretion of the translocon proteins EseB, EseC, and EseD and effector protein EseJ, as revealed by SDS-PAGE and Coomassie blue staining. Complementation of the Δ*esaG* or Δ*esaH* strain restored their secretion profile to that of the WT strain (Fig. 3A). This indicates that EsaG shares the same phenotype as EscE or EsaH, and each is essential for the secretion of T3SS middle and late substrates.

But how do these three proteins influence each other? To investigate, the intracellular steady-state protein level of EsaG, EsaH, or EscE was examined in the WT and Δ*esaG*, Δ*escE*, and Δ*esaH* mutant strains. The EsaH antibody detected a band of ~10 kDa from the WT strain or Δ*esaG* strain and failed to detect a band from the Δ*escE* strain or Δ*esaH* strain. This indicates that EsaH can be specifically probed with the EsaH antibody, and expression of EscE is the premise for a successful detection of EsaH. The EscE antibody detected a band of ~10 kDa from the WT strain or Δ*esaG* strain and failed to detect a band from the Δ*escE* strain, indicating its specificity. EscE was barely detected from the Δ*esaH* strain, which indicates that the steady-state protein level of EscE is largely dependent on the intracellular EsaH. The EsaG antibody detected a band (~10 kDa) only in the cell lysates of the WT strain, but not in those from the Δ*esaH*, Δ*escE*, or Δ*esaG* strain (Fig. 3B). This indicates that both EscE and EsaH are necessary for the steady-state EsaG in *E. piscicida*. The possibility of in-*trans* transcription links among *escE*, *esaH*, and *esaG* was excluded by a real-time PCR assay (data not shown). Therefore, the posttranslational links among them might be involved.

### EsaH and EscE are mutually required for their stability and that of EsaG.

The stabilities of EsaG, EsaH, and EscE in the absence of one another were determined. Given the low efficiency of the EsaG antibody in probing the intracellular EsaG, we incorporated a 3×FLAG tag at the N terminus of chromosomal *esaG*, obtaining WT 3×FLAG::*esaG*, Δ*escE* 3×FLAG::*esaG*, and Δ*esaH* 3×FLAG::*esaG* strains. The three strains were grown in Dulbecco’s modified Eagle’s medium (DMEM) supplemented with chloramphenicol (Cm) to inhibit protein synthesis, and the steady-state protein levels of EscE, EsaH, and EsaG were examined at 60-min intervals until 360 min after chloramphenicol treatment. As shown in Fig. 3C, while the EscE, EsaH, and EsaG protein levels remained unchanged in the WT strain after chloramphenicol treatment, the steady-state protein levels of EsaH and EsaG in the Δ*escE* strain sharply decreased, as did the steady-state protein levels of EscE and EsaG in the Δ*esaH* strain. These results indicate that both EscE and EsaH are essential for maintaining the stability of EsaG, while EscE and EsaH are mutually required for their stability.

### Crystal structure of the EscE-EsaG-EsaH complex.

**FIG 4:**
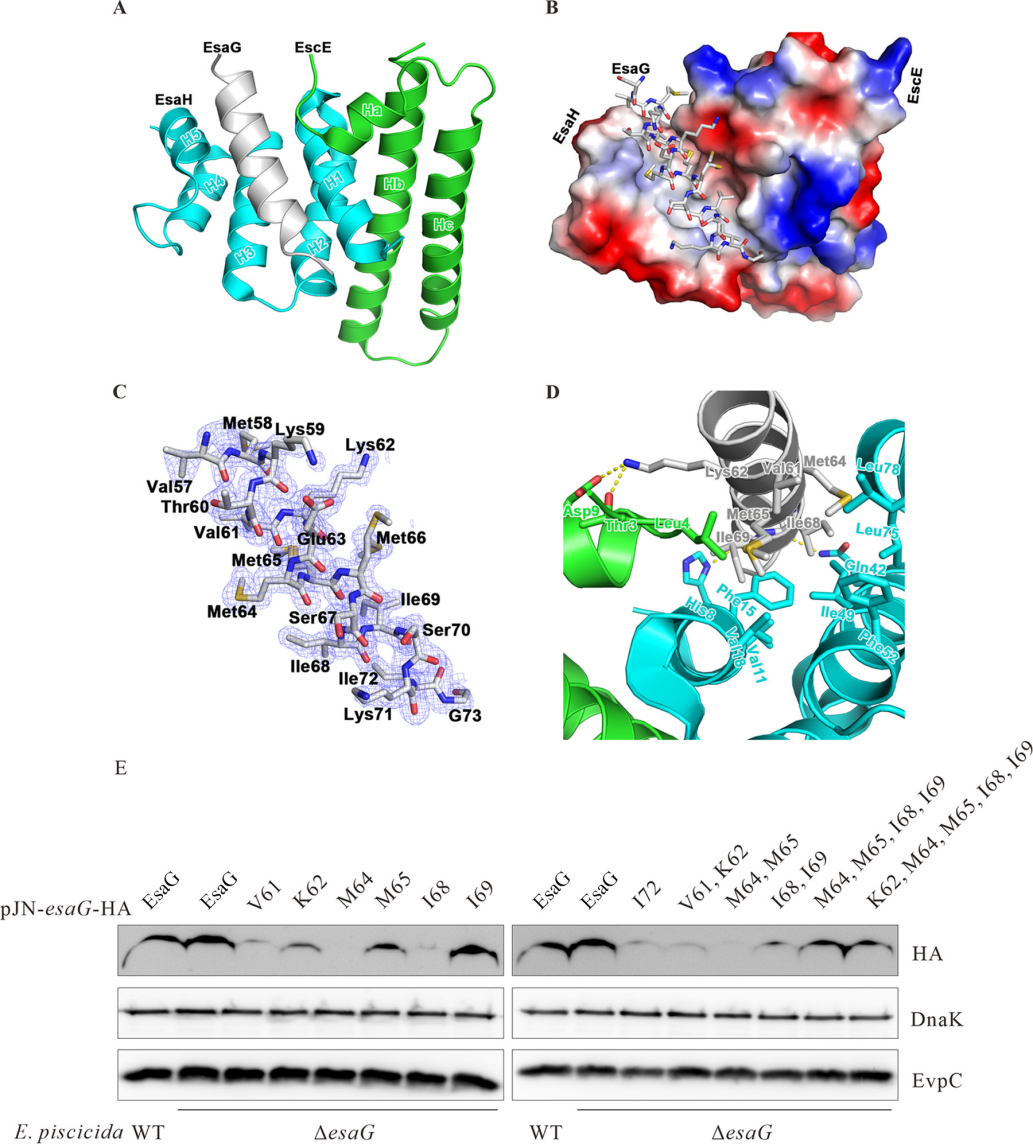
Structure of the EsaG-EsaH-EscE complex. (A) Stereo view of the EsaG-EsaH-EscE complex (cartoon), with the secondary structures labeled. EsaG, EscE, and EsaH are colored in gray, green, and cyan, respectively. (B) Electrostatic surface potential of the EsaG binding site of the EsaH-EscE complex, which is shown on the electrostatic surface, with EsaG shown as sticks, colored as in panel A; (C) 2*F*_o_ − *F*_c_ electron density map (contoured at a level of 1.0 σ) for EsaG in the EsaG-EsaH-EscE complex. The refined model of EsaG is superimposed on the electron density map and shown as the gray stick. The residues are labeled. (D) Closeup view of the EsaG-EsaH-EscE interaction interface, with the hydrogen bonding interactions denoted by yellow dashed lines. Six of the seven hydrophobic residues of EsaG (gray) that were mutated to Ala in this study are shown. (E) Screening of amino acids related to the stability of EsaG. Shown are the results from analysis of the effect of EsaG mutations on its stability in *E. piscicida* from which *esaG* was chromosomally deleted. Pellets from equal amounts of each strain were probed with HA and DnaK antibodies. DnaK, a chaperone protein of *E. piscicida*, and EvpC, a protein secreted through the T6SS, were used as loading controls.

To understand how EscE collaborates with EsaH to stabilize EsaG and to ensure the secretion of EsaG, we determined the crystal structure of the EscE-EsaG-EsaH complex by molecular replacement at a resolution of 1.8 Å, which has been refined to an *R*_work_/*R*_free_ of 20.4%/23.2% with good geometry (Table 2). The calculated electron density map allowed the unambiguous tracing of most of the EscE (2 to 72 aa) and EsaH (4 to 80 aa). However, only the C-terminal region of EsaG (56 to 73 aa) could be observed in the electron density map, and it adopts an α-helical conformation that complexes with EsaH and EscE (Fig. 4A). Notably, H5, an additional helix, was brought out at the C-terminal region of EsaH, and the five-helix bundle formed a groove to grasp EsaG^56–73^ (Fig. 4B). Additionally, the helix Ha of EscE also interacted with EsaG^56–73^. Upon close inspection of these ternary complex interactions, the EsaH residues Val11, Phe15, and Val18 of H1, Ile49 and Phe52 of H3, Leu75 and Leu78 of H5, and Leu4 of EscE were observed to form a hydrophobic pocket, which stabilized the EsaG anchor residues Val61, Met64, Met65, Ile68, Ile69, and Ile72 (Fig. 4C). Moreover, the hydrogen bonds between Lys62 of EsaG and Thr3/Asp9 of EscE anchored the EsaG to EscE, and the hydrogen bonds formed by Ile68 and Ile69 of EsaG together with Gln42 and His8 of EsaH anchored the EsaG to EsaH (Fig. 4D). Taken together, the helices H1 to H5 of EsaH adopt a superhelical scaffold, creating a concave surface and a highly hydrophobic platform that “grasps” the C-terminal helix of EsaG^56–73^. Both EscE and EsaH maintain the carboxyl terminus of their cognate fiber-forming partner EsaG in a coiled-coil interaction.

### Key amino acids related to the stability of EsaG.

Hydrophobic interaction plays a key role in the maintenance of the mechanical infrastructure. The hydrophobic residues aligned along the polymerization domain of the type III secretion needle-forming protein are responsible for its structural integrity and provide key interactions that ensure its robust nature (26). Hydrophobic interaction also plays an important role in maintaining the conformation of the heterotrimer structure. According to the interaction information obtained from the structure of the EscE-EsaG-EsaH complex, individual amino acid substitutions in EsaG were constructed, and their effect on the stability of EsaG was investigated. Targeting the C-terminal region of EsaG by alanine replacement influenced the stability of EsaG. Substitution of M64, V61/K62, or M64/M65 led to a stark degradation of EsaG. Among them, M64 played a pivotal role in stabilizing EsaG, as the protein level of EsaG M64 was the lowest among all the EsaG single-site mutation strains. Interestingly, the protein level of EsaG partially decreased upon point mutation of either V61 or K62, while it sharply decreased upon their double mutation, indicating a cooperative effect of these two sites. Besides, M65, I68, and I72 moderately influenced the stability of EsaG. However, the steady-state levels of EsaG M64/M65/I68/I69 or EsaG K62/M64/M65/I68/I69 were higher than that of EsaG M64/M65, EsaG I68/I69, or EsaG K62 (Fig. 4E). It is speculated that the simultaneous mutation of these multiple sites might have changed the conformation of the ternary complex, thus strengthening the interaction among the other amino acids, which resulted in a new balance.

### Both EsaH and EscE are secreted in a T3SS-dependent manner.

**FIG 5:**
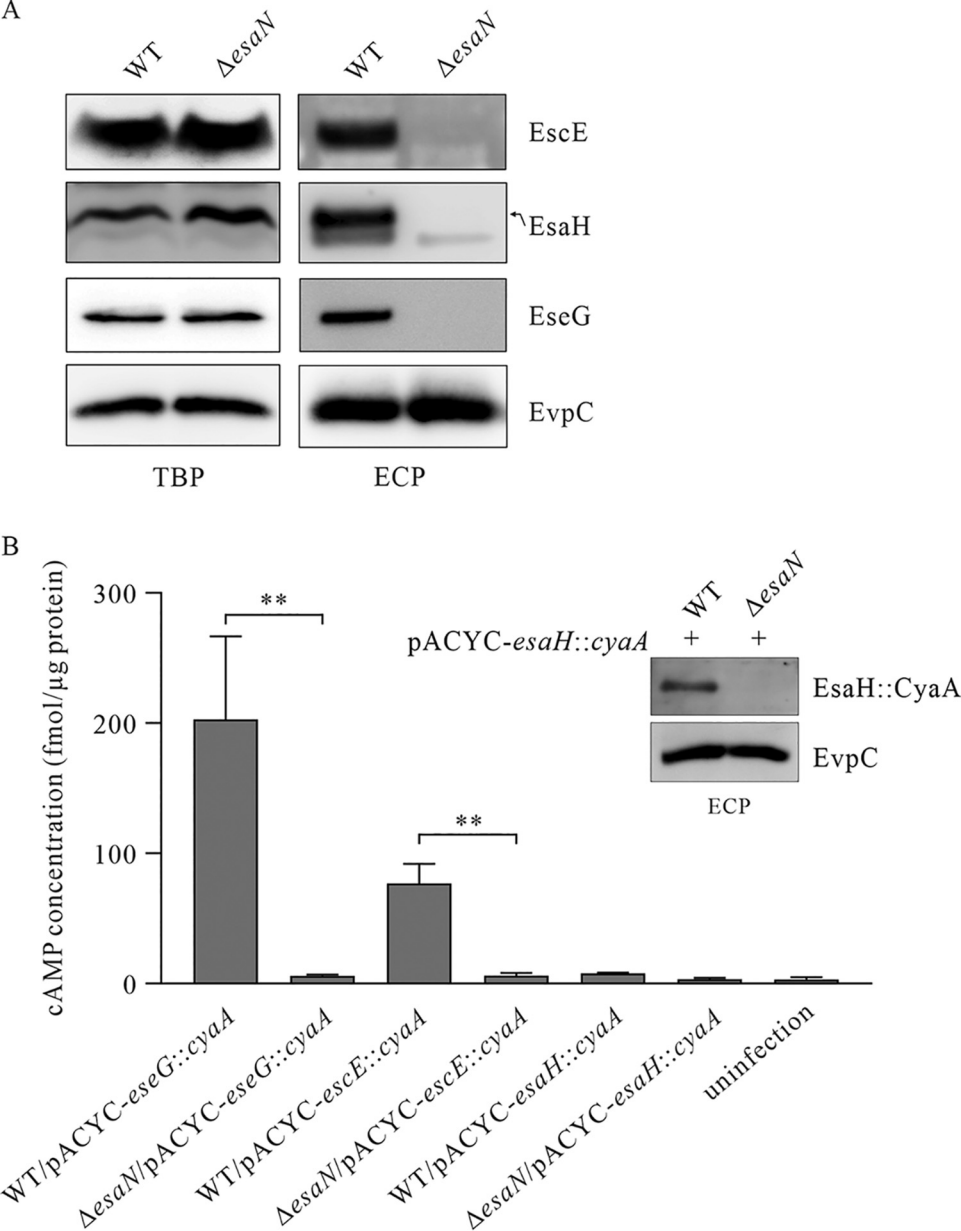

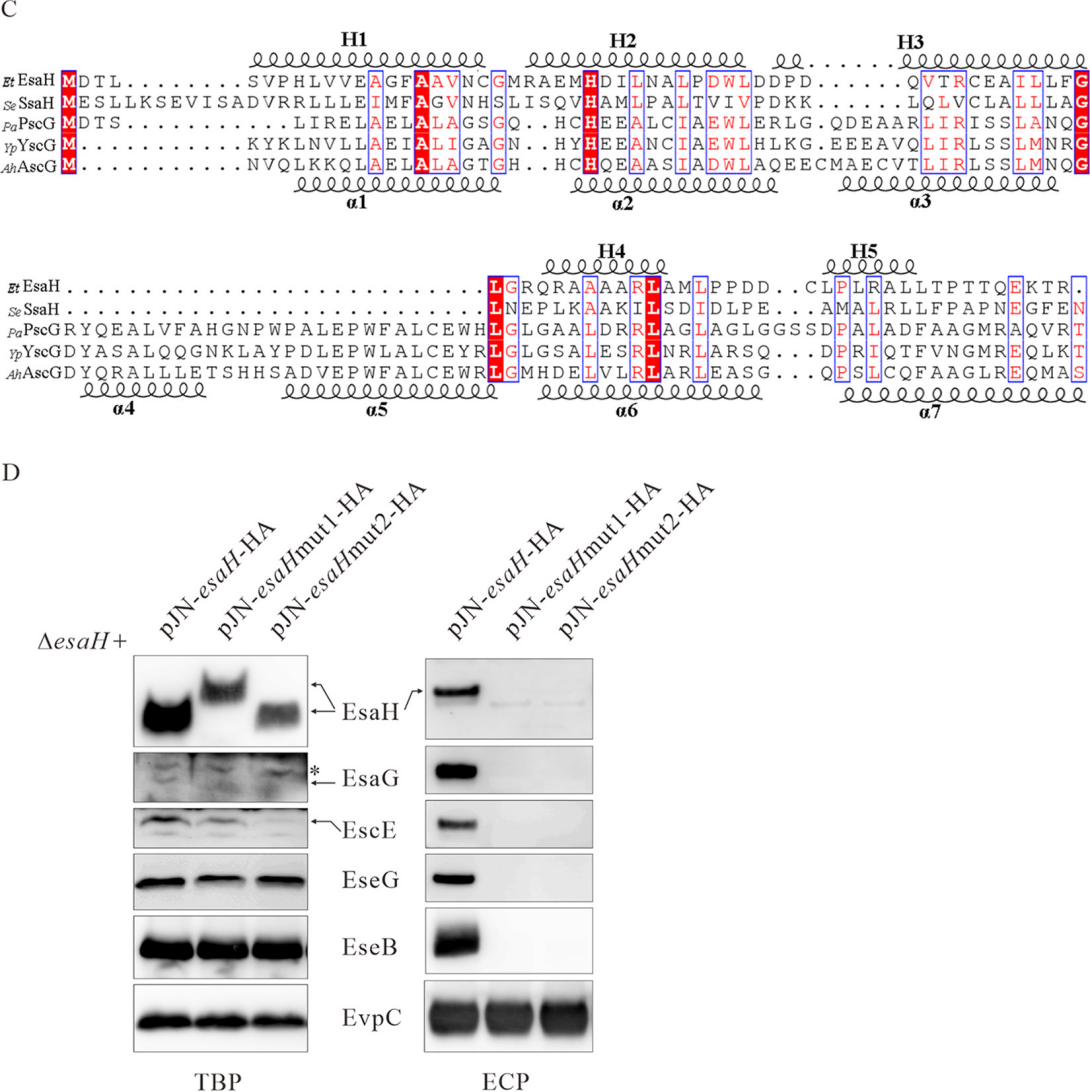
Secretion of EsaH is related to the α4 and α5 helices it lacks and also to its N-terminal sequence. (A) EsaH is secreted in a T3SS-dependent manner. Total bacterial proteins (TBPs) and extracellular proteins (ECPs) from similar amounts of the wild-type strain and T3SS mutant strain (Δ*esaN* strain) were probed with EsaH, EscE, EseG, and EvpC antibodies. EvpC, a protein secreted by the type VI secretion system but not by T3SS, was used as a loading control. (B) EsaH cannot be translocated into host cells. J774A.1 cells were infected with the indicated *E. piscicida* WT strain or Δ*esaN* strain carrying pACYC-*eseG*::*cyaA*, pACYC-*escE*::*cyaA*, and pACYC-*esaH*::*cyaA*, and intracellular cAMP levels were determined at 4 h postinfection, as described in Materials and Methods. Means ± SD from one representative experiment are shown. **, *P* < 0.01. Inset: EsaH::CyaA can be secreted in a T3SS-dependent manner. Secretion of EsaH::CyaA from the WT/pACYC-*esaH*::*cyaA* strain and Δ*esaN/*pACYC-*esaH*::*cyaA* strain was probed with anti-EsaH and anti-EvpC antibodies. EvpC was used as a loading control. (C) Multiple alignment of *E. piscicida* EsaH (GenBank no. ADM40922.1) with its homologs—SsaH from S. enterica subsp. *enterica* (GenBank no. BCH84983.1), PscG from P. aeruginosa (GenBank no. NP_250411.1), YscG from Y. pestis (GenBank no. WP_011901822.1), and AscG from A. hydrophila (GenBank no. ABF70178.1). Seven α helices were found in PscG, YscG, or AscG, and α-helix 4 and α-helix 5 were absent in EsaH or SsaH. The secondary structures of EsaH and PscG are shown at the top and bottom of the graph, respectively. The conserved residues are boxed. (D) Expression and secretion of EsaH, EscE, EsaG, EseB, and EseG from Δ*esaH* strain overexpressing EsaHmut1 or EsaHmut2. Total bacterial pellets or extracellular proteins from the indicated *E. piscicida* strains were immunoblotted for EsaH, EscE, EsaG, EseB, EseG, and EvpC. The immunoblotting data shown are a representative image from three independent experiments.

EscE is required to stabilize EsaH, and it can be secreted through the T3SS (28, 29). But will EsaH also be secreted upon secretion of EscE? To investigate, similar amounts of extracellular proteins (ECPs) and total bacterial proteins (TBPs) of wild-type and Δ*esaN* strains were probed with anti-EsaH, anti-EscE, anti-EseG and anti-EvpC antibodies. EsaN is an ATPase, energizing the secretion of T3SS substrates (4). EvpC, a type VI protein, was used as a loading control. Similar amounts of EsaH, EscE, and EseG were detected in the TBPs of the WT or Δ*esaN* strain; however, the three were only probed from the ECPs of the WT strain, and not from that of the Δ*esaN* strain (Fig. 5A). This indicates that EsaH can also be secreted through the active T3SS.

EscE is translocated into host cells in a T3SS-dependent manner (28, 29), but can EsaH also be translocated? CyaA fusions have been used in many bacteria, including *Edwardsiella*, *Yersinia*, and *Xanthomonas*, to successfully monitor the translocation of bacterial T3SS effectors (28, 32–35). To investigate, pACYC-*esaH*::*cyaA* was constructed, which expresses a chimeric protein, EsaH::CyaA. If the fusion protein is translocated into the host cell, CyaA will convert ATP into cAMP in the presence of the eukaryotic cell cytoplasmic protein calmodulin. J774A.1 cells were infected with the WT/pACYC-*esaH*::*cyaA* strain, the Δ*esaN*/pACYC-*esaH*::*cyaA* strain, the WT/pACYC-*escE*::*cyaA* strain, the Δ*esaN*/pACYC-*escE*::*cyaA* strain, the WT/pACYC-*eseG*::*cyaA* strain, and the Δ*esaN*/pACYC-*eseG*::*cyaA* strain. The intracellular cAMP levels at 4 h postinfection (hpi) were measured as the readout of the translocation of EsaH::CyaA, EscE::CyaA, or EseG::CyaA. As shown in Fig. 5B, the cAMP levels in both WT-infected and Δ*esaN* mutant-infected cells were similar to that of the uninfected control for EsaH::CyaA. The cAMP levels in WT-infected cells were 202.99 ± 63.57 fmol/μg of host protein for EseG::CyaA (positive control) and 76.89 ± 14.91 fmol/μg of host protein for EscE::CyaA (positive control) when the cAMP level of EseG::CyaA or EscE::CyaA was less than 6.0 fmol/μg of host protein in cells infected with the Δ*esaN* strain (negative controls). These results indicate that, different from EscE, which is translocated into host cells in a T3SS-dependent manner, EsaH is not translocated into J774A.1 cells. Next, we investigated whether the failure in EsaH::CyaA translocation is due to its incapability of being secreted. To investigate, extracellular proteins (ECPs) of the WT/pACYC-*esaH*::*cyaA* or Δ*esaN*/pACYC-*esaH*::*cyaA* strain were harvested before being probed with anti-EsaH and anti-EvpC antibodies. EvpC was used as a loading control. It was found that EsaH-CyaA was secreted from the WT but not Δ*esaN* background (Fig. 5B, inset).

Taken together, both EsaH and EscE are secreted in a T3SS-dependent manner, while different from EscE, EsaH fails to be translocated.

### Secretion of EsaH is related either to its N-terminal sequence or the α4 and α5 helices it lacks.

Secondary structure alignment of the T3SS needle chaperones found seven helices from YscG of Y. pestis, AscG of A. hydrophila, and PscG of P. aeruginosa, but only five from EsaH of *E. piscicida* and SsaH of S. enterica: i.e., both EsaH and SsaH lack the α4 and α5 helices (Fig. 5C). To learn whether the absence of these two α helices accounts for the secretion of EsaH, PscG_α4–5_ was inserted into the *esaH* gene, obtaining pJN-*esaH*_α1–3_*-pscG*_α4–5_-*esaH*_α4–5_-HA (pJN-*esaH*mut1-HA). Moreover, as the secretion signal of T3SS substrates is always located in their N-terminal regions, 1 to 25 aa of EsaH were swapped with 1 to 25 aa of PscG, obtaining pJN-*pscG*_1–25 aa_*-esaH*_26–88 aa_-HA (pJN-*esaH*mut2-HA). pJN-*esaH*-HA, pJN-*esaH*mut1-HA, and pJN-*esaH*mut2-HA were introduced into the Δ*esaH* strain, and their expression was induced with l-arabinose. It was observed that insertion of the α4 and α5 helices of PscG into EsaH moderately decreased the steady-state protein level of EscE, while swapping the N-terminal 25 aa with that of PscG starkly decreased EscE, meanwhile, the intracellular EsaG was undetectable under both conditions. The steady-state protein level of EseG, EseB, or EvpC in each strain did not change with the expression of the chimeric EsaH (Fig. 5D, left panel). Secretion of EsaH, EsaG, EscE, EseG, and EseB was detected from the Δ*esaH/*pJN-*esaH*-HA strain, but not from strains expressing any of the chimeric EsaH, when equal amounts of proteins were loaded per lane, as evidenced by EvpC (Fig. 5D, right panel). These data indicate that incorporation of the α4 and α5 helices of PscG into EsaH or swapping the N-terminal portion of EsaH might have interfered with its interaction with EscE or EsaG, therefore crippling the secretion of EsaH, EsaG, EscE, the T3SS translocon, and effectors.

## DISCUSSION

In the absence of EscE, the secretion of EseB, EseC, EseD, or EseJ was obstructed. EscE tightly controls the secretion of T3SS middle and late substrates according to its steady-state protein level inside *E. piscicida* (28), but how EscE controls T3SS secretion in *E. piscicida* remains an enigma. In this article, we provide the first structural evidence that EscE and EsaH are the cochaperones of the T3SS needle protein EsaG. EscE delicately controls the secretion of middle and late substrates of the T3SS through stabilizing EsaG. Moreover, for the first time, we provide evidence that T3SS needle chaperones can be secreted.

Secretion of the T3SS translocon or effectors is obstructed when the T3SS needle chaperones are disrupted in EPEC, Y. pestis, or S. enterica serovar Typhimurium (16, 36, 37). Consistently, in the absence of the T3SS needle protein, secretion of the T3SS translocon and effectors is also obstructed (26, 38, 39). Recently, SsaE was reported to regulate cellular SsaH levels to control the secretion of early substrates of the T3SS needle SsaG and rod SsaI before promoting the secretion of the translocon, thereby demonstrating a chaperone-mediated switch in secretion from early to middle substrates of Salmonella SPI2 (17). In *E. piscicida*, EscE binds with EsaH and forms an EscE-EsaH complex. In the absence of EscE, the transcriptional level of *esaH* remains unchanged (data not shown), while no EsaH can be detected, emphasizing the fact that EscE is stabilizing EsaH. Moreover, in this study, we demonstrated that EsaH is the main chaperone that binds and stabilizes EsaG. Five helices of EsaH create a concave surface and a highly hydrophobic platform that “grasps” the C-terminal helix of EsaG^56–73^. The EscE-EsaH complex maintains the carboxyl terminus of the proteins’ cognate fiber-forming partner EsaG in a coiled-coil interaction, thereby stabilizing EsaG and ensuring its proper secretion. Secretion of EsaG leads to the secretion of T3SS middle and late substrates. In this study, we revealed that EscE regulates the secretion of T3SS substrates through controlling the steady-state protein level of EsaG, whose secretion initiates the secretion of T3SS middle and late substrates.

Seven helices were detected from PscG, YscG, and AscG by secondary structure analysis, and five from EsaH and SsaH, which lacked the fourth and fifth α helices of their homologs. EsaH can be secreted through the T3SS. Inserting the α4 and α5 helices of PscG between the third and fourth α helices of EsaH decreases the steady-state protein level of the chimeric EsaH inside *E. piscicida* and abolishes the secretion of EsaG or EscE. It is speculated that inserting the α4 and α5 helices into EsaH may destroy the interaction between EsaH and EscE or EsaH and EsaG, thereby impeding the secretion of T3SS middle and late substrates. Moreover, the RMSDs of EsaH/PscG and EsaH/YscG were 3.7 Å and 3.1 Å, respectively, indicating that, unlike PscG and YscG, EsaH may be a novel chaperone. The tetratricopeptide repeat region (TPR) is a structural motif of T3SS needle chaperones, and EsaH may form a partial TPR fold, which enables its secretion.

The EscE of *E. piscicida* can be translocated into host cells, where it interacts directly with mammalian factor Cugbp2 (28, 29). Although secreted, EsaH failed to be translocated. At present, only the chaperone Spa15 of S. flexneri has been reported to be secreted through the T3SS (40). Spa15 is associated in the bacterial cytoplasm with IpaA, IpgB1, and OspC3, and it is necessary for the stability of the T3SS effector IpgB1 and for the secretion of the T3SS effector IpaA (40, 41). Moreover, Spa15 is also translocated through the T3SS and contributes to the intracellular survival of *Shigella* by blocking apoptosis in the infected host cells (42). Whether EscE, EsaH, and Spa15 share some common characteristics in their structures that enable their secretion awaits further study.

The structure of the EscE-EsaG-EsaH complex shows that EsaH is the major binding partner of EsaG, EsaH and EscE form a hydrophobic pocket to bind the C-terminal region of EsaG, and this companionship prevents the degradation of EsaG. The hydrophobic pocket formed by EsaH and EscE mainly stabilizes the EsaG anchor residues Val61 and Met64. M64 plays a pivotal role in stabilizing EsaG, and V61 and K62 coefficiently stabilize EsaG to a similar extent to M64. Besides EsaH, EscE also interacts with EsaK, as revealed by the yeast two-hybrid assay results. In the absence of EsaK, the steady-state protein levels of EscE, EsaH, or EsaG are not disrupted, while the translocon or effectors fail to be secreted (unpublished data). Hence, we speculate that EsaK might interact with the EscE-EsaG-EsaH complex and contribute to the secretion of EsaG. Moreover, EsaK shares 19% similarity with SsaK (26). SsaK is the homolog of OrgB, and OrgB is one of the components of the sorting platform in Salmonella SPI1-T3SS (9). It is possible that the EscE-EsaG-EsaH complex might be docked at the sorting platform of *E. piscicida* T3SS through the interaction between EscE and EsaK, facilitating the secretion of EsaG, EscE, and EsaH.

Type III secretion occurs in a hierarchical manner (43): proteins involved in the assembly of the needle complex are secreted first, followed by the translocon and effectors. Early substrates are not injected into host cells (44, 45). In this work, we have proven that EsaH can be secreted through the T3SS but failed to be translocated; thereby, EsaH could be an early T3SS substrate of *E. piscicida*.

Salmonella stimulates gastroenteritis in humans and typhoid-like fever in mice. Salmonella SPI2 is switched on in response to the acidic pH inside the Salmonella-containing vacuole and is required for the establishment of systemic infection (46). The T3SS of *E. piscicida* has almost a full set of genes that are homologous to Salmonella SPI2 (47). By resolving the crystal structure of EscE-EsaG-EsaH, our study also sheds light on the needle assembly of Salmonella SPI2.

## MATERIALS AND METHODS

### Bacterial strains.

The Edwardsiella piscicida PPD130/91 strain used in this study was previously known as Edwardsiella tarda PPD130/91 (1, 48). The *E. piscicida* strains and plasmids used in this study are described in Table 3. *E. piscicida* strains were grown at 25°C in tryptic soy broth (TSB) (BD Biosciences) or in Dulbecco’s modified Eagle’s medium (DMEM) (Invitrogen) under a 5% (vol/vol) CO_2_ atmosphere to activate T3SS. When required, appropriate antibiotics were supplemented at the concentrations of 12.5 μg/mL colistin (Col) (Sigma), 34 μg/mL chloramphenicol (Cm) (Amresco), 15 μg/mL tetracycline (Tet) (Amresco), and 50 μg/mL gentamicin (Gm) (Amresco).

**TABLE 3:** Strains and plasmids used in this study

Strain or plasmid	Description and/or genotype^*a*^	Reference or source
Strains		
*E. piscicida*		
PPD130/91	Wild type, Km^s^ Col^r^ Amp^s^ (LD_50_,10^5.0^)	48
Δ*esaH* mutant	PPD130/91, in-frame deletion of *esaH*_1–69 aa_	This study
Δ*esaG* mutant	PPD130/91, in-frame deletion of *esaG*_1–73 aa_	This study
Δ*escE* mutant	PPD130/91, in-frame deletion of *escE*_1–74 aa_	28
WT 3×FLAG::*esaG*	PPD130/91, chromosomal expression of 3×FLAG-*esaG*, Amp^r^ Km^r^	This study
Δ*esaH* 3×FLAG::*esaG* mutant	Δ*esaH*, chromosomal expression of 3×FLAG-*esaG*, Amp^r^ Km^r^	This study
Δ*escE* 3×FLAG::*esaG* mutant	Δ*escE*, chromosomal expression of 3×FLAG-*esaG*, Amp^r^ Km^r^	28
WT/pACYC-*eseG*::*cyaA*	PPD130/91 with pACYC-*eseG*::*cyaA*	28
Δ*esaN*/pACYC-*eseG*::*cyaA* mutant	Δ*esaN* with pACYC-*eseG*::*cyaA*	28
WT/pACYC-*escE*::*cyaA*	PPD130/91 with pACYC-*escE*::*cyaA*	28
Δ*esaN*/pACYC-*escE*::*cyaA* mutant	Δ*esaN* with pACYC-*escE*::*cyaA*	28
WT/pACYC-*esaH*::*cyaA*	PPD130/91 with pACYC-*esaH*::*cyaA*	This study
Δ*esaN*/pACYC-*esaH*::*cyaA* mutant	Δ*esaN* with pACYC-*esaH*::*cyaA*	This study
Δ*esaH/*pJN-*esaH-*HA mutant	Δ*esaH* with pJN-*esaH-*HA	This study
Δ*esaH/*pJN-*esaH*mut1-HA mutant	Δ*esaH* with pJN-*esaH*_α1–3_*-pscG*_α4–5_-*esaH*_α4–5_-HA	This study
Δ*esaH/*pJN-*esaH*mut2-HA mutant	Δ*esaH* with pJN-*pscG*_1–25 aa_-*esaH*_26–88_ *_aa_-*HA	This study
E. coli		
DH5α	α complementation	Stratagene
BL21(DE3)/pLysS	F^−^ *ompT hsdS* (r_B_^−^ m_B_^−^) *gal dcm*(DE3)*tonA* pLysS(Cm^r^)	Invitrogen
Plasmids		
pMD18-T	Cloning vector, Amp^r^	TaKaRa
pRE112	Suicide plasmid, *pir* dependent, Cm^r^ *oriT oriV sacB*	49
pRE-Δ*esaH*	pRE112 with *esaH* flanking fragments	This study
pRE-Δ*esaG*	pRE112 with *esaG* flanking fragments	This study
pET-21a		Novagen
pACYC-*esaH*::*cyaA*	pACYC184 with *cyaA* fused to C terminus of *esaH*	This study
pJN-105	Arabinose-inducible gene expression vector *araC*-P_BAD_, Gm^r^	51
pKD46	Red helper plasmid, Amp^r^	60
pKD4	Template plasmid with FLP recognition target site, Km^r^	60
pJN-*esaH-*HA	pJN-105 with *esaH-*HA	This study
pJN-*esaH*mut1-HA	pJN-*esaH*_α1–3_*-pscG*_α4–5_-*esaH*_α4–5_-HA	This study
pJN-*esaH*mut2-HA	pJN-*pscG*_1–25 aa_-*esaH*_26–88 aa_*-*HA	This study
pJN-*esaG*-HA	pJN-105 with *esaG-*HA	This study
pJN-*esaG* V61-HA	pJN-105 expressing EsaG with site mutation of V61A	This study
pJN-*esaG* K62-HA	pJN-105 expressing EsaG with site mutation of K62A	This study
pJN-*esaG* M64-HA	pJN-105 expressing EsaG with site mutation of M64A	This study
pJN-*esaG* M65-HA	pJN-105 expressing EsaG with site mutation of M65A	This study
pJN-*esaG* I68-HA	pJN-105 expressing EsaG with site mutation of I68A	This study
pJN-*esaG* I69-HA	pJN-105 expressing EsaG with site mutation of I69A	This study
pJN-*esaG* I72-HA	pJN-105 expressing EsaG with site mutation of I72A	This study
pJN-*esaG* V61 K62-HA	pJN-105 expressing EsaG with site mutations of V61A and K62A	This study
pJN-*esaG* M64 M65-HA	pJN-105 expressing EsaG with site mutations of M64A and M65A	This study
pJN-*esaG* I68 I69-HA	pJN-105 expressing EsaG with site mutations of I68A and I69A	This study
pJN-*esaG* M64 M65 I68 I69-HA	pJN-105 expressing EsaG with site mutations of M64A, M65A, I68A, and I69A	This study
pJN-*esaG* K62 M64 M65 I68 I69-HA	pJN-105 expressing EsaG with site mutations of K62A, M64A, M65A, I68A, and I69A	This study

a Col, colistin; Amp, ampicillin; Tet, tetracycline; Cm, chloramphenicol. Superscripts: r, resistance; s, sensitivity.

### Construction of knockout mutants and plasmids.

The *esaH* or *esaG* gene was deleted from the chromosome of *E. piscicida* PPD130/91 by *sacB*-based allelic exchange using a method previously described (49, 50). The mutants obtained were verified by sequencing and immunoblotting. All primers used are listed in Table 4. The complementing plasmids pJN*-esaH-*HA and pJN*-esaG-*HA are derivatives of pJN105 (51), which carries the *esaH* or *esaG* gene under the control of l-arabinose-inducible *araBAD* promoter. The fourth and fifth α-helix sequences of the *pscG* were inserted into *esaH* to obtain pJN-*esaH*_α1–3_*-pscG*_α4–5_-*esaH*_α4–5_-HA (pJN-*esaH*mut1-HA). In a similar way, pJN-*pscG*_1–25 aa_*-esaH*_26–88 aa_-HA (pJN-*esaH*mut2-HA) was constructed. The plasmids acquired were verified by DNA sequencing before being transferred into *E. piscicida* strains and their expression was verified by immunoblotting.

**TABLE 4:** Oligonucleotides used in this study

Designation	Nucleotide sequence
*esaG*-for	ATGGTACCGCTTATCGGCTGGAATGTTGTTGTA
*esaG*-int-rev	CACGGTAAGGAGCCTATTGCCATGGATACGCTGTCAGTTCC
*esaG*-int-for	ATAGGCTCCTTACCGTGTTTAGCC
*esaG*-rev	ATGGTACCCTCAGTCAGGAGCACCTCATGC
*esaG-*check-for	TCGTTGTTGACATTGCCAGAGGTG
*esaG-*check-rev	TCATCACCTCTGGCAATGTCAACAA
*esaH*-for	ATGGTACCGATTGATTTCGGGGCTGTATT
*esaH*-int-rev	CAAAATAGGGTAATGCCGACGACTGCCTGCCGCTGC
*esaH*-int-for	GGCATTACCCTATTTTGCTGATGATGC
*esaH*-rev	ATGGTACCAGCCTGACCCTGGAGTGTCAACGGA
*esaH-*com-for	CCGGAATTCCGGAGTACTCCGTCATGATCGGCT
*esaH-*com-rev	TGCTCTAGAGCATCAGAGGCTAGCATAATCAGGAACATCATACGGATATCGCGTTTTCTCCTGAGTGGT
*esaG-*com-for	CCGGAATTCCGGTGCTTTCGGCGTCACTTT
*esaG-*com-rev	TGCTCTAGAGCATTAGAGGCTAGCATAATCAGGAACATCATACGGATAGCCCTATTT
EsaG-2F	GGATCCAACATTGAGGATATCGTCTCTCAG
EsaG-33F	GGATCCGATCCGCAGGCCATGCT
6His-EsaG-33F	CCATGGGTCATCACCATCATCACCACGGATCCGATCCGCAGGCCATGCT
EsaG-73R	CTCGAGTTACCCTATTTTGCTGATGATGC
EsaH-2F	CCATGGGTGATACGCTGTCAGTTCCTCA
EsaH-88R	CTCGAGTCATCGCGTTTTCTCCTGAG
EscE-1F	CCATGGGTATGCCCACCCTAACGCATCT
EscE-73R	CTCGAGTTAGCCTTGGTGCAGCGTCC
*esaH*-*cyaA*-F	CGGGGTACCCCGTCATGATCGGCTACC
*esaH*-*cyaA*-R	GGAAGATCTTCGCGTTTTCTCCTGAGT
3×FLAG-*esaG*-for	GCCAGCCTTCACCACCGCGCCGGCTAAACACGGTAAGGAGCCTATGTGTAGGCTGGAGCTGCTTC
3×FLAG-*esaG*-rev	GCGGGAAACCTGATTGCCCAGCTGAGAGACGATATCCTCAATGTTTTTATCGTCGTCATCTTTGTAGTCGATATCATGATCTTTATAATCACCGTCATGGTCTTTGTAGTCCATATGAATATCCTCCTTA

### Immunoblotting analysis.

*E. piscicida* extracellular proteins (ECPs) and bacterial lysates (total bacterial proteins [TBPs]) were prepared as described by Zheng and Leung (52). They were loaded onto a 12% NuPAGE gel for electrophoresis in morpholineethanesulfonic acid (MES) running buffer (Invitrogen) or loaded onto 16% Tricine SDS-PAGE as described by H. Schägger (53). For immunoblotting analysis, proteins transferred onto polyvinylidene difluoride (PVDF) membrane (Millipore) were probed with rabbit antibodies against HA (1:3,000) (Sigma), EseG (1:3,000) (7), EvpC (1:5,000) (52), EscE (1:1,000) (28), EseB (1:3,000) (54), EsaG (1:1,000), or EsaH (1:1,000). The EsaH antibody was raised in mice against EsaH protein purified from E. coli BL21(DE3), the EsaG antibody was raised in rabbit against aa 18 to 31 (SNDAHSVITSGNVN), which was conjugated with keyhole limpet hemocyanin, and the antisera obtained were purified using the specific peptide as the ligand (Genscript, China). To probe EscE, EsaH, or EsaG, proteins transferred onto PVDF membrane (0.22-μm pore) were fixed in 2.5% glutaraldehyde for 1 h before being subjected to blocking and immunoblotting.

### Yeast two-hybrid assay.

The yeast two-hybrid assay was carried out using a GAL4 DNA-binding domain-encoding bait vector (pGBKT7) and a GAL4 activation domain-encoding prey vector (pGADT7) in the yeast strain AH109, as described by Liu et al. (30). All fusion constructs were made as full-length fusions. The interaction between human lamin C bait fusion (pGBKT7-Lam) and simian virus 40 (SV40) prey fusion (pGADT7-T) (Clontech) was used as a negative control, and the interaction between murine p53 bait fusion (pGBKT7-53) and pGADT7-T served as a positive control.

### Protein expression, purification, and gel filtration chromatography.

The *escE* gene was cloned into a glutathione *S*-transferase (GST) fusion protein expression vector pGEX-6P-1 (GE Healthcare) and *esaH* into pET-28b. The two plasmids obtained were cotransformed into E. coli BL21(DE3), and cultures were grown at 37°C to an optical density at 600 nm (OD_600_) of 0.6 to ~0.8, before induction with 0.1 mM isopropyl-β-d-thiogalactopyranoside (IPTG) (Sigma, USA) for 16 h at 20°C. The harvested cells were sonicated in buffer A (400 mM NaCl, 50 mM Tris-HCl [pH 8.0], 10% glycerol). Cell lysate was centrifuged at 18,000 × *g* for 1 h at 4°C to collect supernatant, which was incubated with glutathione Sepharose 4 FastFlow beads (GE Healthcare) at 4°C for 3 h. The proteins on beads were digested with 3C protease. The protein complex was further purified by size exclusion chromatography (Hiload 16/600 Superdex 75 prep grade) in buffer B (25 mM Tris-HCl [pH 8.0], 150 mM NaCl) as the running buffer. Purified protein was analyzed by SDS-PAGE. The peak fraction was pooled and concentrated to 15 mg/mL. Selenomethionine-labeled EsaH/EscE was purified the same way as the unlabeled proteins.

To copurify the three proteins, pETDuet-1-His_6_-*esaG*^33–73^-*esaH* and pET-28b-*escE* were cotransformed into E. coli BL21(DE3), and the three proteins were purified using Ni-nitrilotriacetic acid (NTA) beads (GE Healthcare). The proteins were eluted from the beads with Ni-binding buffer containing 0.25 M imidazole, 400 mM NaCl, and 50 mM Tris-HCl (pH 8.0). Proteins were further purified on HiLoad 16/600 Superdex 75 prep-grade (GE Healthcare) gel filtration column in buffer B. The proteins purified were analyzed by SDS-PAGE. The peak fraction was pooled and concentrated to 19 mg/mL.

### Crystallization and data collection.

Crystals of the EscE-EsaH complex were obtained in hanging drops in 0.1 M KSCN, 0.1 M Tris (pH 8.4), 23% PEG monomethyl ether 2000, supplemented with a final concentration of 3% (wt/vol) 1,6-hexanediol, and were developed for 2 days at 16°C. The EscE-EsaG^33–73^-EsaH complex crystal was developed with 3.3 M NaCl and 0.1 M each KH_2_PO_4_ and Na_2_HPO_4_ (pH 6.2) at 16°C. All the diffraction data were collected at Shanghai Synchrotron Research Facility (SSRF) on beamline BL17U, BL18U, or BL19U, integrated, and processed with the HKL2000 program suite and XDS packages (55). The structure of EscE-EsaH was solved by single-wavelength anomalous diffraction (SAD) method. The complex structure of EscE-EsaG^33–73^-EsaH was solved by molecular replacement with the newly solved EscE-EsaH as the search model using the program Molrep (56). Refmac5 (57) was used to ensure the presence of the C-terminal region of EsaG.

All of the structures were iteratively built with COOT (58) and refined with the PHENIX program (59). Parameters on data collection and structure refinement are summarized in Table 2. All figures were generated using the program PyMOL (http://www.pymol.org/).

### Stability of EsaG with site mutation.

According to the structure information on the interaction between EsaG and EsaH/EscE, EsaG V61A, K62A, M64A, M65A, I68A, I69A, I72A, V61A K62A, M64A M65A, I68A I69A, M64A M65A I68A I69A, and K62A M64A M65A I68A I69A site mutations were obtained through gene synthesis (Genscript, China), and the DNA fragments were ligated into pJN105. The plasmids obtained were introduced into the Δ*esaG* strain, respectively. In parallel, the wild-type copy of *esaG* was inserted into pJN105, and the resulting plasmid was introduced into the Δ*esaG* strain, which was used as the control for the EsaG stability assay. The steady-state protein levels of EsaG obtained from the strains described above were probed.

### CyaA translocation assay.

The CyaA translocation assay was conducted on J774A.1 macrophage cells, as previously described (28), and cyclic AMP (cAMP) levels were assayed by using an enzyme-linked immunoassay (ELISA) according to the instruction of the manufacturer (Arbor Assays).

### Protein stability assay.

The *E. piscicida* WT 3×FLAG::*esaG*, Δ*escE* 3×FLAG*::esaG*, and Δ*esaH* 3×FLAG*::esaG* strains were constructed as reported previously (60), subcultured into DMEM at 1:100, and maintained at 25°C with 5% (vol/vol) CO_2_ until an OD_540_ value of ~0.4 was reached. Then, the culture was supplemented with chloramphenicol at a final concentration of 200 μg/mL, and bacterial cultures were sampled every 60 min until 6 h posttreatment. These samples were subjected to immunoblotting with anti-FLAG (EsaG), anti-EscE, anti-EsaH, or anti-DnaK antibody. DnaK, a chaperone protein, was used as a loading control. The protein stability experiment was repeated at least three times, and representative images are shown.

### Statistical analysis.

The results were analyzed using GraphPad Prism 6. Statistical significance was calculated using the two-tailed Student's *t* test. Results are expressed as the mean ± standard deviation (SD).

### Data availability.

The atomic coordinates of *E. piscicida* PPD130/91 EscE-EsaH and EscE-EsaG^33–73^-EsaH have been deposited in the Protein Data Bank under PDB ID 7Y6B and 7Y6C.
