# Supplementary material for: Secreted in a Type III Secretion System-Dependent Manner, EsaH and EscE Are the Cochaperones of the T3SS Needle Protein EsaG of Edwardsiella piscicida
Source: mBio. 2022 Jul 21;13(4):e01250-22. doi: 10.1128/mbio.01250-22 (PMC9426511; doi:10.1128/mbio.01250-22)
Supplement: FIG S1 [file mbio.01250-22-s0001.doc]

Supplementary

sFig.1


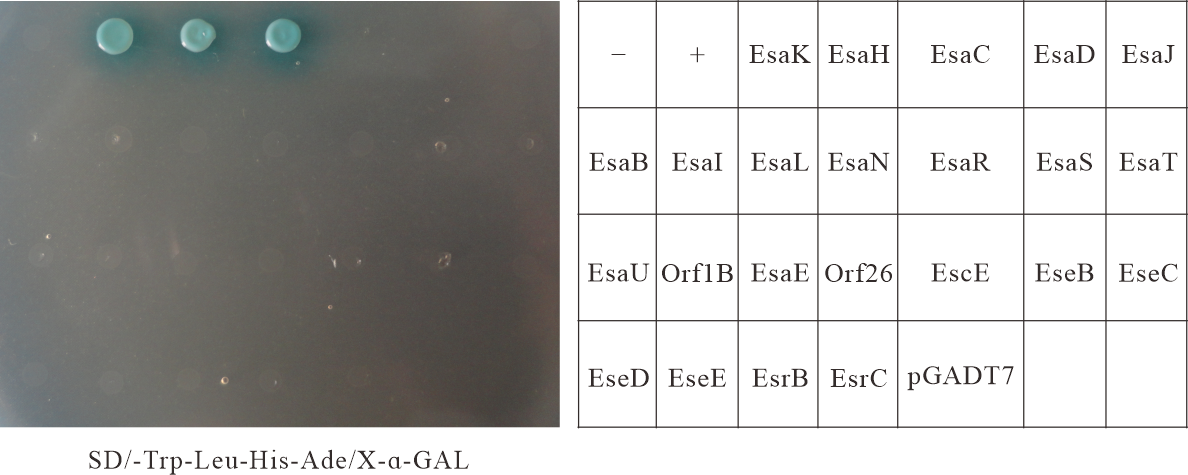


Figure legend: EscE interacts with EsaH and EsaK as revealed by yeast two-hybrid assay. Yeast two-hybrid results using high stringency SD lacking Trp, Leu, His and Ade were shown (left panel). Yeast strains transformed with pGBKT7-*escE* plus pGADT7-*esaH* or pGBKT7-*escE* plus pGADT7-*esaK* were able to grow in the selective medium as blue colonies like the positive control (+, transformed with pGADT7-T plus pGBKT7-53). Yeast strain transformed with pGADT7-T plus pGBKT7-Lam was the negative control (-). The other yeast strains transformed with pGBKT7-*escE* plus pGADT7 inserted with *esaC*, *esaD*, *esaJ*, *esaB*, *esaI*, *esaL*, *esaN*, *esaR*, *esaS*, *esaT*, *esaU*, *orf1B*, *esaE*, *orf26*, *escE*, *eseB*, *eseC*, *eseD*, *eseE*, *esrB*, or *esrC* failed to grown on the SD/-Trp-Leu-His-Ade plate.
